# Comparison of Measurements of Autoantibodies to Glutamic Acid Decarboxylase and Islet Antigen-2 in Whole Blood Eluates from Dried Blood Spots Using the RSR-Enzyme Linked Immunosorbent Assay Kits and In-House Radioimmunoassays

**DOI:** 10.1155/2010/173652

**Published:** 2010-06-03

**Authors:** Anders Persson, Charlotte Becker, Ida Hansson, Anita Nilsson, Carina Törn

**Affiliations:** ^1^Unit for Diabetes and Celiac Disease, Department of Clinical Sciences, Lund University, Wallenberg Laboratory, Entrance 46, University Hospital MAS, 205 02 Malmö, Sweden; ^2^Department of Clinical Chemistry, University Hospital MAS, 205 02 Malmö, Sweden; ^3^Unit for Diabetes and Celiac Disease, Department of Clinical Sciences, Lund University, Clinical Research Center (CRC), University Hospital MAS, 205 02 Malmö, Sweden

## Abstract

To evaluate the performance of dried blood spots (DBSs) with subsequent analyses of glutamic acid decarboxylase (GADA) and islet antigen-2 (IA-2A) with the RSR-ELISAs, we selected 80 children newly diagnosed with type 1 diabetes and 120 healthy women. DBSs from patients and controls were used for RSR-ELISAs while patients samples were analysed also with in-house RIAs. The RSR-ELISA-GADA performed well with a specificity of 100%, albeit sensitivity (46%) was lower compared to in RIA (56%; *P* = .008). No prozone effect was observed after dilution of discrepant samples. RSR-ELISA-IA-2A achieved specificity of 69% and sensitivity was lower (59%) compared with RIA (66%; *P* < .001). Negative or low positive patients and control samples in the RSR-ELISA-IA-2A increased after dilution. Eluates from DBS can readily be used to analyse GADA with the RSR-ELISA, even if low levels of autoantibodies were not detected. Some factor could disturb RSR-ELISA-IA-2A analyses.

## 1. Introduction

Sampling of blood as dried blood spots (DBSs) for clinical use is currently used for such diverse diseases as congenital metabolic disorders, diabetes, and HIV infections [1–4]. There are a number of advantages gained when using the DBS-technique with subsequent elution concerning ease of collection, transportation/storage, small blood volumes, and minimal invasiveness compared with serum samples. DBS-technique facilitates sampling procedures since capillary sampling can be used. The capillary blood sampling requires less skill and fewer supplies, it can even be used by diabetes patients at home if they are used to measuring their own blood glucose levels. DBS samples can easily be mailed since there is no risk of leakage. Both transportation and short time storage can be done at room temperature [5, 6]. In many countries, all newborn babies are screened for phenylketonuria, galactosemia, congenital hypothyroidism, and other metabolic disorders using dried blood spots, indicating that this technique is suitable for large scale analyses [7–9]. The DBS-samples can be used for both genetic factors (DNA) [10] and proteins (enzymes and antibodies) [5, 11].

Five islet autoantibodies are known to characterize type 1 diabetes, namely, islet cell antibodies (ICA), insulin autoantibodies (IAA), glutamic acid decarboxylase antibodies (GADA), islet antigen-2 antibodies (IA-2A), and antibodies against the beta cell specific zink-transporter (ZnT8A) [12]. The first four of these are well characterized and several workshops have been undertaken to standardize the measurement of these autoantibodies in serum [13–15]. Both in-house RIAs and RSR-ELISA kits are well established for analyses of GADA as well as for IA-2A. The RSR-ELISA kits show high performance for both GADA and IA-2A in serum samples [15] and Ca^2+^-treated plasma can also be used [16, 17]. In DASP 2005, both RSR-ELISA-GADA kits and RSR-ELISA-IA-2A kits achieved high sensitivity and specificity [15]. For RSR-ELISA-GADA kits (*n* = 7) sensitivity varied from 84% to 94% and specificity from 97% to 99% for serum samples. For RSR-IA-2A-ELISA kits, sensitivity ranged from 64% to 68% and specificity from 98% to 100% for serum samples. In DASP 2005, our in-house RIA-GADA assay gave a sensitivity of 76% and a specificity of 91% for serum samples, and our in-house RIA-IA-2A assay gave a sensitivity of 72% and a specificity of 100%. GADA and IA-2A have been analysed in whole blood eluates with RIA assays with high performance [18, 19]. 

In this study, we wanted to test if GADA and IA-2A can be analysed from whole blood eluates with RSR-ELISAs. If these assays show high performances, the measurements of GADA and IA-2A using ELISA have a potential to be applied in large screening programs for identifying individuals at risk for type 1 diabetes.

## 2. Materials and Methods

### 2.1. Subjects

Dried blood spots (DBS) were obtained as EDTA-blood spotted onto filter forms (Parajett, Parajett AB, Landskrona, Sweden, with filters from Schleicher and Schuell, Dassel, Germany) and air-dried before transportation to the laboratory. A minimum of 60 *μ*L blood was needed to fill the marked circles on the filters.

The study population consisted of a random selection of children with newly diagnosed type 1 diabetes (*n* = 80; median age 10 yrs; range 2–18; M/F = 1.29) and healthy control women were obtained from five Maternity Clinics in our region as described [20] (*n* = 120; median age 32 yrs; range 19–44). The patient's (DBSs) had been stored at room temperature for a median of 56 days (range 8–150) and (DBSs) from controls had been stored for a median of 18 days (range 5–57) before punching with subsequent elution was performed.

All samples were collected under informed consent, as approved by the Ethical Committee at Lund University, Lund.

### 2.2. Sample Preparation

Discs with a diameter of 6 mm were punched out with a special punching device (Wallac Delfia dried blood spots puncher prod no. 1296-071, Wallac, Turku, Finland). Four discs were punched out from each specimen into separate wells. A total of 80 *μ*L of TBST-buffer (150 mmol/L NaCl, 20 mmol/L Tris, pH 7.4, 0.15% Tween20, 0.1% BSA) was added to each well. Samples were left on a plateshaker (Delfia plateshaker 1296-003, Wallac, Turku, Finland) at 500 rpm, at +4°C overnight. Next morning, whole blood eluates were spun down (1 min, 1500 × g, Labofuge 400, Heraeus, Langerbold, Germany). Whole blood eluates were pooled into an Eppendorf-micro tube and spun at 10000 × g for 10 min to remove cell debris.

### 2.3. Assays

#### 2.3.1. RSR-ELISA Assays

RSR-ELISA kits for GADA (GDE/96) and IA-2A (IAE/96) (RSR Ltd, Pentwyn, Cardiff, UK) were used for analyses of GADA and IA-2A. The assays were performed according to the instructions from the manufacturer, except that whole blood eluates were used in equal amounts as recommended for serum. Optical density was read on an ELISA platereader (E-max, Multical platereader, Molecular Devices Corporation, Menlo Park, CA, USA) at 450 nm, with software Multicalc (Perkin-Elmer, Waltham, MA, USA).

Standards were calibrated against the WHO reference NIBSC (97/550) for the GADA assay [21]. High values (>250 WHO Units/ml) were replaced with 250 for statistical and clinical evaluations. The cut-off level was set to 5 WHO Units/ml for GADA, which is the lowest standard concentration and also the recommended cut-off by the manufacturer for serum samples. 

Also for IA-2A, standards were calibrated against the NIBSC (97/550). High values (>400 WHO Units/ml) were replaced with 400 for statistical and clinical evaluations. The cut-off level was set to 15 WHO Units/ml for IA-2A, which is the lowest standard concentration and also the recommended cut-off by the manufacturer for serum samples. Duplicate sampling including the whole preanalytic procedure was performed in ten subjects for GADA and IA-2A. The coefficient of variation (CV) was calculated as the ratio between standard deviation and mean value for duplicates. The median value of these observations was 6.3% (range 0.76–13) for GADA in the range of 5.0–46.4 WHO Units/ml. Interassay variation for the same samples in two repetitions was a median 7.7% (range 1.8%–40%). The CV for IA-2A was a median 5.0% (range 0.77–15) in the range 15–25 WHO Units/ml. Interassay variation in two repetitions was a median 46% (range 25%–88%). 

Samples with a high level of antibodies (GADA or IA-2A) in the in-house RIA, but low in the ELISA were diluted to reveal if this finding could be validated or was due to the prozone effect. The prozone effect is well-known to interfere with titers for ICA [22, 23].

#### 2.3.2. In-House RIA for GADA and IA-2A

Aliquots of 30 *μ*L of whole blood eluates were obtained using the procedure described in *Sample preparation* and were added into wells with 30 *μ*L of ^35^S-radiolabelled antigen (GAD65 or IA-2) and incubated overnight on a plateshaker (500 rpm) at +4°C. Next morning, plates were spun for 1 min 1500 × g. Duplicates of 50 *μ*L of the antibody-antigen-complex-solution were added to 50 *μ*L of 20% rProtein A Sepharose Fast Flow (Amersham Biosciences, Uppsala, Sweden) and incubated for 90 minutes at +4°C on a plateshaker (500 rpm). Excess antigen was removed by repeated washing of plates (8 times with cold TBST-buffer) using a special washing device (Multiscreen vacuum washer, Millipore, Bedford, MA, USA). Plates were air-dried for 30 min, before addition of 50 *μ*L of scintillation liquid (Optiphase Supermix scintillation fluid, PerkinElmer Life Sciences, Boston, MA, USA) to each well. The radioactivity was measured in a beta counter (Microbeta counter, PerkinElmer Life Sciences, Boston, MA, USA).

Logaritmic standard curves were used for the GADA assay and IA-2A assay. Our laboratory uses the WHO-standard as local standard. Samples above 50 WHO-Units/ml were considered as positive in the GADA assay as were samples above 10 WHO-Units/ml in the IA-2A assay. These cut-off limits were defined using previous results from healthy individuals. A GADA level of 500 WHO Units/ml and an IA-2A level of 250 WHO Units/ml were considered as endpoints for samples analysed with RIA and were not diluted further. 

The median CV for duplicates was 3.5% (range 0–15; *n* = 29) for GADA and interassay variation was 10.1% (*n* = 29) for a sample of 35 WHO Units/ml. For another sample of 96 WHO Units/ml, median CV was 3.1 % (range 0–11; *n* = 29) and interassay variation was 8.7% (*n* = 29).

The median CV for duplicates was 3.7% (range 0–10: *n* = 30) for IA-2A and interassay variation was 7.8% (*n* = 30) for a sample of 24 WHO Units/ml. For another sample of 155 WHO Units/ml the median CV was 2.7% (range 0–11: *n* = 24) and interassay variation was 9.7%.

#### 2.3.3. Statistical Analysis

Results are reported as median, interquartile range and minimum and maximum since not normally distributed. If *P*-values were <.05 a significant difference was accepted. Wilcoxons signed rank test was used to test for differences in repeated measurements in continuos variables.

McNemars test was used to test for differences in binominal paired observations (positive and negative results).

## 3. Results

### 3.1. RSR-ELISA-GADA Assay Characteristics for Measurements from Whole Blood Eluates

The threshold of 5.0 WHO-Units/ml corresponded to a specificity of 100% and a sensitivity of 46% (37/80; Table 1). A total of 8 samples showed a concentration of 250 WHO Units/ml or higher, these samples were diluted and showed final concentrations of 222-2460 WHO-Units/ml.

### 3.2. Comparisons of RSR-ELISA-GADA and an In-House RIA Using Patient's Samples

**Figure 1:**
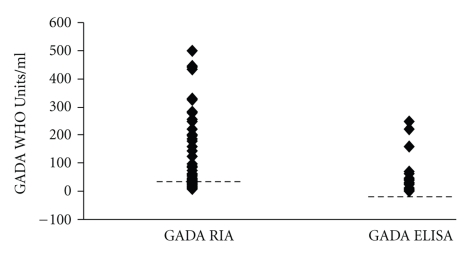
GADA levels detected by both ELISA and RIA assays. Cut off levels are indicated by dashed lines.

**Figure 2:**
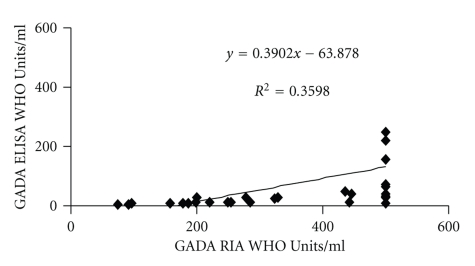
GADA levels correlated in patient's samples positive in both the RSR-ELISA and an in-house RIA (*n* = 37; *r*_*s*_ = 0.82; *P* < .01).

The RSR-ELISA-GADA achieved lower sensitivity 46% (37/80) compared with the in-house RIA 56% (45/80; *P* = .008; Figure 1). All samples (*n* = 37) that were positive in the RSR-ELISA-GADA were also positive in the in-house RIA. The discordant samples were all low level positive samples in the in-house RIA (*n* = 8; range 53–125 WHO-Units/ml; threshold 50 WHO-Units/ml). Twelve samples were high when analysed with the in-house RIA (range 198–≥500 WHO-Units/ml) but relatively low in the RSR-ELISA (range 7.3–46 WHO-Units/ml). These samples were checked for prozone effect by dilution but levels were similar after dilution (range 10–52; *P* = .81). There was a correlation in GADA levels in patient's samples found to be positive in both assays (*n* = 37; *r*_*s*_ = 0.82; *P* < .01; Figure 2). Moreover, GADA levels analysed in double-positive samples were higher in the in-house RIA (*n* = 37; median ≥500 WHO Units/ml; interquartile range 235–≥500) compared to GADA levels analysed with ELISA (*n* = 37; median 32 WHO Units/ml; interquartile range 11–189;  *P* < .001).

### 3.3. RSR-ELISA-IA-2A Assay Characteristics for Measurements from Whole Blood Eluates

Using the cut-off of 15 WHO-Units/ml the specificity was 69% (83/120) and the sensitivity was 59% (47/80; Table 1). A total of 16 samples were 400 WHO Units/ml or higher, after dilution the final concentrations of those samples were ranging from 365 to 3430 WHO-Units/ml.

### 3.4. Comparisons of RSR-ELISA-IA-2A and an In-House RIA Using Patient's Samples

**Figure 3:**
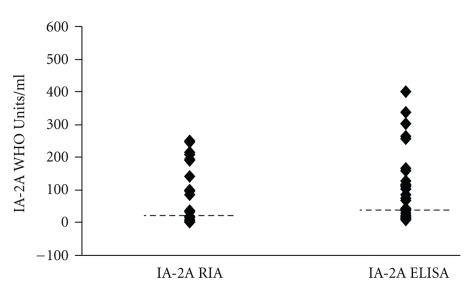
IA-2A levels detected by both ELISA and RIA assays. Cut off levels are indicated by dashed lines.

**Figure 4:**
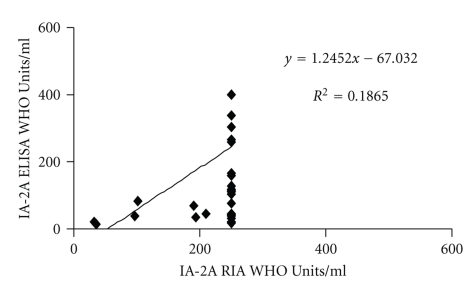
A weak correlation was found in IA-2A levels in positive samples both in the RSR-ELISA and an in-house RIA (*n* = 42; *r*_*s*_ = 0.52; *P* < .01).

The RSR-ELISA-IA-2A achieved lower sensitivity 59% (47/80) compared with the in-house RIA 66% (53/80; *P* < .001; Figure 3). Five low level positive samples in the RSR-ELISA-IA-2A (17–35 WHO Units/ml) were negative in the in-house RIA. Eleven samples were positive only in the in-house RIA (range 17–≥250), six of these samples were 214 WHO Units/ml or higher. We re-analysed two discordant samples that were high level positive in the RIA but negative in the ELISA (with sample material left) and another four samples that were high in the IA-2A RIA (*n* = 6; range 193–≥250 WHO-Units/ml) but low in the RSR-ELISA (*n* = 6; range 11–36 WHO-Units/ml). After dilution IA-2A levels increased in all samples (*n* = 6; range 113–516 WHO-Units/ml; *P* = .028) when analysed in the RSR-ELISA-IA-2A. Four patient's samples that were all negative in the in-house RIA, three of whom were low level positive in the RSR-ELISA and one that was negative (*n* = 4; range 11–35 WHO-Units/ml) were diluted and reanalysed in the RSR-ELISA and also in this case, levels increased (*n* = 4; range 82–888 WHO-Units/ml). Furthermore, 10 control samples found to be clearly negative in the RSR-ELISA (*n* = 10; range 8–13 WHO-Units/ml) were also diluted and reanalysed and also in this case levels were higher when diluted (range 29–127 WHO-Units/ml; *P* = .005). A total of 42 samples were positive in both assays. There was a weak correlation between IA-2A double positive samples analysed with ELISA and RIA (*n* = 42; *r*_*s*_ = 0.52; *P* < .01; Figure 4). Nevertheless, for IA-2A double positive samples, levels were similar in the in-house RIA (*n* = 42; median ≥250 WHO Units/ml; interquartile range ≥250 –≥250) compared with ELISA (median 162 WHO Units/ml; interquartile range 44–≥400; *P* = .76).

### 3.5. Combined Sensitivity for GADA and IA-2A with In-House RIA and RSR-ELISAAs

Combining the results from GADA and IA-2A measurements with in-house RIAs increased the sensitivity for detecting type 1 diabetes to 79% (63/80; *P* = .0020). Likewise, combining the results from GADA and IA-2A measurements with RSR-ELISAs increased the sensitivity to 71% (57/80; *P* = .0020) and expected specificity decreased to 69% (83/120).

## 4. Discussion

In this study, we compared in-house RIAs with commercial ELISAs for analyses of GADA and IA-2A from whole blood eluates both qualitatively and quantitatively. We found that specificity was excellent (100%) for the RSR-ELISA-GADA, while sensitivity was lower (46%) compared with the in-house RIA (56%). Discordant samples that were negative in the RSR-ELISA-GADA were low level positive in the in-house RIA. Moreover, GADA levels were lower in the RSR-ELISA in samples found to be positive in both assays. Samples found to have high GADA levels in the in-house RIA, but low in the RSR-ELISA were reanalysed in dilution but levels did not increase. These findings indicate that samples that are positive in the RSR-ELISA-GADA are concordant with measurements in the in-house RIA. However, the RSR-ELISA failed to detect low level GADA. The lower frequency and lower levels of GADA positive samples can be due to interference of haemoglobin or some other factor in the whole blood. The possibility to measure very high level autoantibodies has little importance in the routine clinical laboratory but can be of interest in intervention studies aimed to decrease levels of autoantibodies [24].

The specificity was very low for RSR-ELISA-IA-2A (69%) and also the sensitivity was lower for the RSR-ELISA-IA-2A (59%) compared with the in-house RIA (66%). Among the discordant patient samples, most were high level positive in the in-house RIA. When two of the discordant samples and also four other patient's samples found to be low level positive or negative in the RSR-ELISA-IA-2A were reanalysed in dilution in the RSR-ELISA, levels increased significantly. Also when ten control samples were reanalysed in dilution, IA-2A levels increased. We believe that haemoglobin or some other factor in whole blood interfered with the measurement of the antibodies, either via a direct binding to the antigen or antibodies or through a colour shift that affected the optical density, even though we have not fully examined the impact of haemoglobin in this study. It must be borne in mind that these commercial kits are recommended for analyses of autoantibodies in serum. One limitation with our study is that we have not analysed paired serum and DBS samples from patients and controls. However, a similar set of patient serum samples have shown excellent performance for the RSR-ELISA-GADA and IA-2A in our hands [17]. Both our in-house RIAs for GADA and IA-2A achieved high sensitivities (76% and 72%) at excellent specificities in DASP 2005 [15]. RSR-ELISAs for GADA and IA-2A were recently established at the Department of Clinical Chemistry, UMAS, Malmö and have not been subjected to international standardisation. Another possible limitation with our study is that our reference population consisted of only women. Nonetheless, GADA has been shown to be more frequently found in women with type 1 diabetes than in men and also levels are higher in women [25, 26]. Samples were drawn prior to delivery in these women used as the control population. Pregnancy could decrease immune response, but GADA has been detected at higher frequency among women with gestational diabetes compared with the general population [27]. Therefore, we assume that we have not limited the ability to detect GADA positive subjects in this reference population using the cut-off limits recommended by the manufacturer. 

We have not done a specific study on reproducibility over time due to limited amount of specimens. However, GADA and IA-2A autoantibodies are of IgG type [28] as are HIV-antibodies [29]. HIV-antibodies have shown excellent reproducibility for up to six weeks in different storage conditions (room temperature,4°C, −20°C, −70°C, and also 37–70°C) [6]. Total IgE has been also shown to be stable for repeated freeze/thaw cycles [5]. HbA1c, another protein frequently used for analyses with DBS-technique, has shown low (less than 2%) between-day imprecision for both venous and capillary sampling [1]. Furthermore, our findings indicate that strong haemolysis may interfere with analyses of IA-2A using the RSR-ELISA. It is possible that haemoglobin caused the lower sensitivity for the RSR-ELISA-GADA but specificity was excellent in this case. 

In conclusion, the RSR-ELISA can be used for measurement of GADA in whole blood eluates in a reliable manner even if sensitivity is lower compared with an in-house RIA. Some factor could disturb RSR-ELISA-IA-2A analyses.

## Figures and Tables

**Table 1:** Estimation of positivity for GADA and IA-2A. Number of positive and negative patients (80 children newly diagnosed with type 1 diabetes; median age 10; range 2–18 yrs) and controls (120 healthy women; median 32; range 19–44 yrs) from Sweden.

Assay	Positive	Negative
	*Patients (* *n* = 80)	

RSR-ELISA-GADA	37 (46%)	43 (54%)
In-house RIA-GADA	45 (56%)	35 (44%)

	*Controls (* *n* = 120)	

RSR-ELISA-GADA	0 (0%)	120 (100%)
In-house RIA-GADA	NA	NA

	*Patients (* *n* = 80)	

RSR-ELISA-IA-2A	47 (59%)	33 (41%)
In-house RIA-IA-2A	53 (66%)	27 (34%)

	*Controls (* *n* = 120)	

RSR-ELISA-IA-2A	37 (31%)	83 (69%)
In-house RIA-IA-2A	NA	NA
